# Examining determinants of gender attitudes: evidence among Tanzanian adolescents

**DOI:** 10.1186/s12905-020-01057-8

**Published:** 2020-09-10

**Authors:** Tia Palermo, Yekaterina Chzhen, Nikola Balvin, Lusajo Kajula, Tanzania Adolescent Cash Plus Evaluation Team, Tia Palermo, Valeria Groppo, Jacobus de Hoop, Lusajo Kajula, Leah Prencipe, Jennifer Waidler, Johanna Choumert Nkolo, Respichius Mitti, Nathan Sivewright, Koen Leuveld, Bhoke Munanka, Paul Luchemba, Tumpe Mnyawami Lukongo, Aroldia Mulokozi, Ulrike Gilbert, Paul Quarles van Ufford, Rikke Le Kirkegaard, Frank Eetaama

**Affiliations:** 1 Department of Epidemiology and Environmental Health, University at Buffalo (State University of New York), 268F Farber Hall, Buffalo, NY 14214 USA; 2 Department of Sociology, Trinity College Dublin, 3 College Green, Dublin 2 Dublin, Ireland; 3 UNICEF India, Near 73, Near, Lodhi Rd, Lodhi Gardens, Lodhi Estate, New Delhi, Delhi, 110003 India; 4 UNICEF Office of Research – Innocenti, Via degli Alfani 58, 50121 Florence, Italy

**Keywords:** Gender attitudes, Adolescents, Tanzania

## Abstract

**Background:**

The shaping of gender beliefs and attitudes in early adolescence affects the way young people internalize and self-enforce prevalent notions of masculinity and femininity, with lifelong consequences for sexual and reproductive health. This cross-sectional study examines determinants of gender attitudes among some of the poorest and most vulnerable adolescents in Tanzania using an ecological model.

**Methods:**

Data come from baseline interviews with 2458 males and females aged 14–19 years conducted as part of a larger impact evaluation. Structural equation models are used to examine how factors at the community-, household-, and individual-levels influence gender attitudes in the four domains measured by the Gender Equitable Men (GEM) Scale (i.e. violence, sexual relationships, reproductive health and disease prevention, and domestic chores and daily life).

**Results:**

A structural equation model of the four latent domains of the GEM scale regressed on individual, social-interactional and structural level characteristics indicated that secondary school attendance was associated with more equitable gender attitudes, while females held less equitable attitudes than males in the sample. Having had sexual intercourse was associated with more gender equitable attitudes among females, but the reverse was true among males.

**Conclusions:**

Addressing gender inequity requires understanding gender socialisation at the socio-interactional level. As females had more inequitable gender attitudes than males in the study, a special emphasis on highlighting the rights of women to girls should be considered. This study will inform future analysis of programme impacts on gender attitudes and sexual and reproductive health.

## Introduction

Adolescence and the transition to adulthood is not only a key development window of rapid physical, sexual and neurological changes, but also a period where new social roles and power relations begin to manifest. Key decisions and transitions, including relationship formation, sexual debut, pregnancy and marriage, are made during adolescence. These can have lasting impacts on both the individuals who make them and the next generation. These decisions and transitions are influenced by the process of gender socialization which intensifies during adolescence. It is a process in which ‘individuals develop, refine and learn to ‘do’ gender through internalizing gender norms and roles as they interact with key agents of socialization, such as their family, community, social networks and other social institutions’ [1].

Gender socialization differs across societal contexts, communities and families and is shaped by influences from different levels of the socio-ecological framework [1]. At the macro level, influences include socio-economic conditions and patriarchal, political and social structures. At the meso-level, agents of influence include the family, peers and social networks, social institutions, such as the school and religious groups, and the neighbourhood. At the individual level, factors such as sex, ethnicity, cognitive and motivational processes, physical and sexual maturation, and personality all influence how a person may be treated by others and internalize their gender identity [1].

Although adolescence is a time when many gender inequities begin to manifest, it is also a window of opportunity to influence the gender socialization process and communicate healthier, more equitable gender norms [1, 2]. Investments made during this period may lead to benefits with a triple dividend among adolescents today, tomorrow and in future generations [3].

George et al. (2020) highlighted that girls and women are most disadvantaged by structural forms of gender inequality [4], resulting from power relations which determine the organization of societies, the enacting of laws, functioning of economies, and shaping of ideologies [5]. For example, gender norms that prioritize early pregnancy and marriage, as well as women’s roles as caregivers and overseeing domestic responsibilities may impede women’s access to schooling and labor market opportunities. Because norms determine what is valued and acceptable and often privilege what is male over what is female, Geroge et al. (2020) note, they help shape institutions (eg, communities, families, markets), and thus influence health exposures, vulnerabilities, access to services, and outcomes [4].

In Tanzania in particular, gender inequalities are evident in outcomes related to education, livelihoods, property rights and asset ownership, political participation, health, violence, child marriage, [6–8]. According to the World Economic Forum’s Global Gender Gap Report, in Tanzania, gender gaps exist in all dimensions of life. According to this index, where a ratio of 1 indicates gender parity, there exist gender gaps which favour men in terms of economic participation and opportunity (0.676), educational attainment (0.918), health and survival (0.978), and political empowerment (0.245). Moreover, there are high rates of child marriage, particularly among girls, and adolescent childbearing [9]. A recent national study found that men and women in Tanzania are generally not supportive of equality when it comes to day-to-day gender relations, including gender roles in the household, power and decision-making, violence against women, sexuality and reproduction, and attitudes about sexual orientation [6]. These attitudes are often informed by childhood experiences, and women face high levels of intimate partner violence, controlling behaviours by their partners, and forced sexual debut [6].

**Fig. 1:**
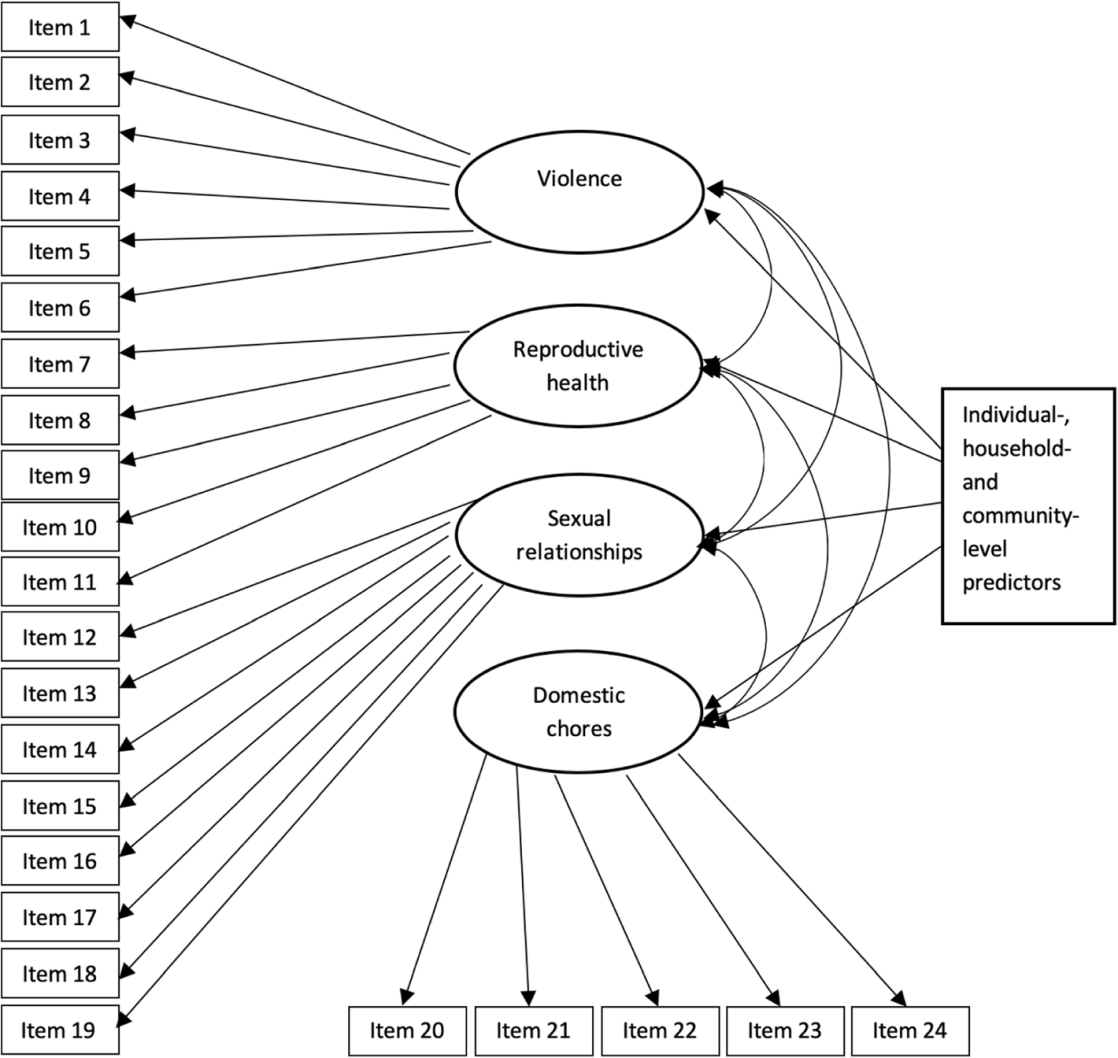
Measurement and structural model

Efforts to design new interventions that aim to influence gender socialization towards improving gender equality must be informed by an in-depth understanding of the individual-, household- and community-level determinants of gender norms and attitudes in the context in which they are implemented. A recent global review examining factors shaping gender attitudes in early adolescence demonstrated how the evidence base on this topic is dominated by studies from higher-income settings such as North America and Western Europe (90% of studies reviewed) [10]. In the current study, we examine individual-, household- and community-level influences on gender attitudes among some of the poorest and most vulnerable adolescents in Tanzania using the socio-ecological framework developed by John et al. [1] (see Fig. 1).

Drawing on this framework, this study aims to test associations between different factors and gender attitudes using a recently adapted tool to measure gender attitudes among young people. Although founded in the literature, the framework is a conceptual one and has not previously been used to examine the determinants of gender attitudes. In this paper we apply the framework in an analysis of survey data in four districts in Tanzania to: a) examine its usefulness for empirical tests; and b) gain a deeper understanding of how micro-, meso- and macro- level factors influence gender attitudes in different contexts. This study helps fill gaps in the evidence from rural, lower-income settings and empirically tests the framework. Furthermore, the results may help inform both the design of new programmes aimed at influencing gender socialization and future analysis of programme impacts on gender attitudes in existing interventions.

## Methods

### Study location

The current study utilizes data collected in the Iringa and Mbeya regions of Tanzania, a country in East Africa with a population of approximately 50 million people [9]. The two regions are located in the Southern Highlands zone, and the cash crops for export produced in both regions include coffee, tea, cocoa and spices. Among women aged 15–29 years, 80 and 77.8% are employed (defined as having done any work in the past 7 days) in Iringa and Mbeya, respectively (compared to 72.3% nationally) [9]. Among men, the percentages are 88.5 and 83.5% in Iringa and Mbeya, respectively (87.5 nationally). The percentage of men with no education is 14.9 and 12.5 in Iringa and Mbeya, respectively; while among women, these percentages are 22.1 and 19.1% in Iringa and Mbeya, respectively. Child marriage is a problem in Tanzania, where 30.5% of girls are married before their 18th birthday. The median age of first sex is 17.6 and 18 for females and males, respectively, in the Southern Highlands and 17.1 and 18.9%, respectively, in the South West Highlands. Moreover, 33% of females aged 15–19 years in Mbeya have already started childbearing, compared to 20% in Iringa [9].

### Participants

Data used in this study come from the baseline survey of an impact evaluation entitled Ujana Salama (‘Safe Youth’ in Swahili) [11]. Data are from a sample of adolescents in Tanzania living in poor households that participate in the national social protection programme called the Productive Social Safety Net (PSSN) [12]. Because we use only baseline data, this is an observational study. However, more information on the overall impact evaluation study design and sampling is provided in the Additional file 1.

The sample includes 130 clusters (communities) from two districts in the Iringa region (Mufindi and Mafinga districts) and two in the Mbeya region (Rungwe and Busokelo districts) of Tanzania. Adolescents were considered eligible if they were 1) living in a PSSN household and 2) between 14 and 19 years of age, and all adolescents meeting these criteria were targeted for interviews. Baseline data collection occurred between April and June, 2017. Of the eligible 6559 adolescents, 2458 completed interviews, and the remaining 4101 did not complete an interview (2960 were subsequently deemed not eligible according to study criteria and 1141 were not interviewed for other reasons).

At the adolescent-level, key outcomes measured included gender attitudes, livelihoods skills and knowledge, economic activities, sexual debut, pregnancy, marriage, school attendance, aspirations, psychosocial wellbeing, violence victimization and perpetration, sexual exploitation, and health and sexual risk-taking behaviours. Household surveys collected information about dwelling characteristics, household composition and demographics (age, education of members), and livelihood activities of the household. Community surveys aimed to assess access to markets, health facilities, schools; prices; village customs surrounding matrilineal v. patrilineal descent, inheritance, and wife inheritance practices; caregiving (who would be expected to take in a child if the parent dies); and shocks.

Interviews were administered by same-sex enumerators in Swahili, face to face, and due to the sensitive nature of several topics, they were conducted in private locations where other household members could not hear what was being discussed. Enumerators inputted responses into SurveyBe software during the interviews.

### Measures

Gender attitudes were measured using an adapted Gender-Equitable Men (GEM) Scale, a version which was originally used with adolescents and young people in Uganda [2]. This adaptation of the scale removed the homophobia sub-scale – as is often the practice in similar settings [13]. Another adaptation of the GEM scale has also been implemented previously in Tanzania, and findings supported applicability for this setting [6]. The resulting scale comprises 24 items assessing support for inequitable gender attitudes across four domains: violence, sexual relationships, reproductive health and disease prevention, and domestic chores and daily life (items listed in Table 2). For the main analysis reported in this study, each item was scored on a 3-point scale (1 = agree, 2 = partially agree and 3 = do not agree; range 24 to 72). We also constructed a continuous GEM scale by dichotomising all items (‘agree’ and ‘partially agree’ v. ‘do not agree’) and summing up the responses. The scale ranged from 0 to 24, with higher values reflecting more equitable attitudes. We used this alternative measure in robustness checks.

Characteristics examined at the individual level included gender, age (14–15 years versus 16–19 years), highest level of completed education (less than secondary versus some secondary or higher), and whether the adolescent had ever had sex (defined as vaginal or anal intercourse). Next, at the household-level (social-interactional level according to Fig. 1), we examined household livelihood activities and asked whether the adolescent specifically participated in any of the following in the prior 7 days: farm work, or livestock herding, household business. Finally, at the community and structural levels, we assessed distance to the nearest daily market (in kilometres), and gender-equitable practices regarding inheritance, including whether a woman can inherit her husband’s land if he dies. In Tanzania, the Village Land Act (1999) empowers villages with land administration, including registration, adjudication, titling and land disputes, and in rural areas (including the study region), customary law is strong [7]. Customary laws are based on the culture and beliefs held by tribes, and there is variation in tribes across the regions in our study. Our study districts in Mbeya are dominated by the Nyakyusa tribe, while the districts in Iringa are mainly Hehe or Kinga tribes who both share similar customary laws. Thus, inheritance practices can vary across our study areas.

### Statistical analysis

We assigned all 24 items to their respective content domains of the GEM scale and tested if indeed the items tapped into the four GEM domains using confirmatory factor analysis (see Fig. 1). To analyse the individual, household and community level predictors of gender equitable attitudes, we used the four latent domains of the GEM scale as dependent variables in a linear structural equation model (*sem* command in Stata 15). We allowed the error terms of the four latent variables (i.e. violence; reproductive health; sexual relationships; and domestic chores) to be correlated with each other. There are two main advantages to this approach as opposed to a regression model with a continuous 24-item GEM scale. First, by modelling the four latent constructs simultaneously we allowed the predictors to have different associations with each domain of gender equitable attitudes. Second, by isolating the unique variances of the 24 items from their shared variances across the four domains we minimized measurement error. We used the predictors described in the measures section above. We allowed the effects of all covariates to vary by region (i.e., Mbeya vs. Iringa) and tested for group invariance.

As a robustness check we regressed the 24-point GEM scale on the same predictors, separately by district, in three specifications that treated the village clustering differently, including (1) adjusting the standard errors for clustering; 2) controlling for village identifiers as fixed effects; and 3) modelling them as random effects (i.e.*,* hierarchical/multilevel modelling).

### Patient and public involvement

Patients were not involved in this study.

## Results

### Descriptive analysis

The analytic sample of the current study comprises 1880 respondents with no missing data on any of the 24 gender attitudes questions. Table 1 shows that the mean GEM score was 12.5 out of 24 in the whole sample. It was 1.2 points lower for females than for males (11.9 vs. 13.1; *p* < 0.05). The majority of the sample came from the Iringa region (60%), were male (55%), and were 16 to 19 years old (61%). Nearly one-third (32%) had completed at least eight grades of education. While nearly one-fifth (19%) of 14–19-year-olds reported having ever had sexual intercourse, the prevalence was significantly lower among 14–15-year-olds than among 16–19-year-olds (3% vs. 29%; *p* < 0.0001). Female 14–19-year-olds were significantly more likely to report having had sex than males (23% vs. 15%, *p* < 0.001). Most respondents lived in households that engaged in farm work (66%), some in households engaging in livestock herding (42%), and a minority (5%) in households which ran a business.

**Table 1:** Characteristics of the working sample (*N* = 1880)

	All	Female	Male
*Individual level*			
Mean GEM scale (0–24)	12.5	11.9	13.1
Gender (%)			
Female	44.7		
Male	55.3		
Age (%)			
14–15 years old	38.9	44.1	34.7
16–19 years old	61.1	55.9	65.3
Highest level of education completed (%)			
Less than secondary	67.8	61.6	72.8
Some secondary or higher	32.2	38.4	27.2
Ever had sexual intercourse (%)			
No	81.2	76.6	85.0
Yes	18.8	23.4	15.0
*Household level*			
Household engaged in farm work (%)			
No	34.5	43.0	27.5
Yes	65.5	57.0	72.5
Household engaged in livestock herding (%)			
No	57.8	67.2	50.1
Yes	42.2	32.8	49.9
Household has a business (%)			
No	95.5	95.4	95.6
Yes	4.5	4.6	4.4
*Village level*			
Women can inherit husband’s land (%)			
No	7.1	5.8	8.1
Yes	92.9	94.2	91.9
Region (%)			
Iringa	59.5	62.5	57.1
Mbeya	40.5	37.5	42.9
Mean distance to nearest daily market (km)	18.9	19.5	18.4

Table 2 shows that female respondents expressed significantly higher levels of support for inequitable gender attitudes in 11 of 24 GEM items. The gender difference was reversed for just one item, in reproductive health: female adolescents were significantly less likely to agree that ‘women who carry condoms on them are easy’ (38% of females vs. 44% of males; *p* < 0.05).

**Table 2:** Levels of inequitable gender attitudes by gender (% agreed)

	Female (*N* = 841)	Male (*N* = 1039)
Item1: There are times a woman deserves to be beaten.	35.7	38.0
Item 2: A woman should tolerate violence in order to keep her family together.	42.4	42.6
Item 3: If someone insults a man he should defend his reputation with force if he has to.	**41.4**	**35.0**
Item 4: It is okay for a man to hit his wife if she will not have sex with him.	29.4	23.7
Item 5: A man using violence against his wife is a private matter that should not be discussed outside the couple.	**43.5**	**33.9**
Item 6: It is alright for a man to beat his wife if she is unfaithful.	**56.6**	**41.7**
Item 7: It is a woman’s responsibility to avoid getting pregnant.	**57.8**	**50.7**
Item 8: A man should be angered/shocked if his wife asks him to use a condom.	40.0	39.6
Item 9: Women who carry condoms on them are easy.	**37.9**	**44.4**
Item 10: Only when a woman has a child is she a real woman.	**62.8**	**50.0**
Item 11: A real man produces a male child.	38.0	35.7
Item 12: It disgusts me when I see a man acting like a woman.	72.2	71.5
Item 13: A woman should not initiate sex.	47.0	48.4
Item 14: You do not talk about sex, you just do it.	35.6	39.8
Item 15: A woman who has sex before she marries does not deserve respect.	51.0	52.7
Item 16: Men need sex more than women do.	38.8	33.4
Item 17: Men are always ready to have sex.	**47.6**	**38.0**
Item 18: A man needs other women, even if things with his wife are fine.	**37.6**	**30.0**
Item 19: It is the man who decides how he wants to have sex.	48.4	43.5
Item 20: Giving the kids a bath and feeding the kids are the mother’s responsibility.	**75.5**	**59.8**
Item 21: A woman’s most important role is to take care of her home and cook for her family.	**79.9**	**67.9**
Item 22: A man should have the final word on decisions in his home.	**69.0**	**63.6**
Item 23: The husband should decide what major household items to buy.	60.5	56.9
Item 24: A woman should obey her husband in all things.	**65.9**	**54.2**

Gender differences significant at *p* < 0.05 are in bold. Standard errors adjusted for clustering by village

### Structural equation modelling

The model mapping four latent factors of the GEM scale fit with the quantitative survey data well even after accounting for the complex design of the survey. All factor loadings and error covariances between the four latent variables were substantial and statistically significant at *p* < 0.001, the standardized root mean squared residual (SRMR) was less than 0.05 (SRMR = 0.04), and the coefficient of determination (CD) was nearly 1 (CD = 0.98). For comparison, a one-factor model offered a worse fit (SRMR = 0.06; CD = 0.86). A four-factor measurement model also offered a good fit to the data in each of the districts (see Table A1 in the Additional file 1). Across the six error covariance’s between the four latent domains, reproductive health and sexual relationships domains were the most highly correlated with each other, and violence and domestic chores the least.

Table 3 shows estimates from a structural equation model of the four latent domains of the GEM scale regressed on individual, household and community level characteristics separately by district. Among adolescents living in Mbeya who reported never having had sex, gender attitudes were significantly less equitable among females compared to males across all four domains of the GEM scale. Among those in Iringa, the gender difference was only significant for the domestic chores domain. There was a positive interaction between gender and having had sex (at *p* < 0.10 in each dimension) in Mbeya (but not in Iringa), indicating that females who had had sex reported more equitable gender attitudes, much closer to that of their male peers. In Mbeya, the effect of having had sex was significantly more negative for males in all four dimensions, but this association did not hold among females.

**Table 3:**
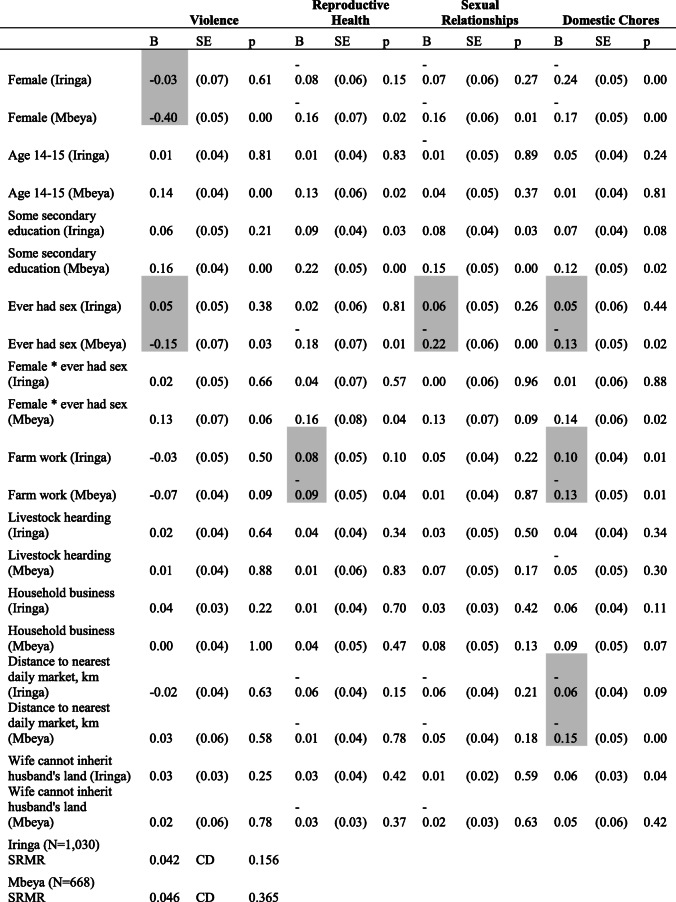
Four-factor structural equation model of gender norms (standardized coefficients)

Notes: SRMR = standardized root mean squared residual

Standard errors are adjusted for clustering by village

Statistically significant differences (*p* < 0.05) by district are shaded. Standard errors adjusted for clustering by village. Factor loadings, variances and co-variances are omitted

In Mbeya, younger adolescents (ages 14–15 years) reported more equitable gender attitudes in the violence and reproductive health domains, as compared to older adolescents in the sample. Those with at least some secondary education reported more equitable gender attitudes in all domains in Mbeya and in all but the violence domain in Iringa.

In Mbeya, farm work was associated with less gender equitable attitudes with respect to violence, reproductive health and domestic chores. In Iringa, farm work was associated with more equitable attitudes in terms of reproductive health and in domestic chores. Greater distance to the nearest market centre was associated with less equitable attitudes with respect to domestic chores in both districts, with a significantly more negative effect in Mbeya. In Iringa, adolescents expressed more equitable gender attitudes with respect to domestic chores and daily life in communities with inheritance norms that discriminated against women.

### Robustness checks

To determine if the loss of 24% of the sample due to listwise deletion of missing data across the 24 GEM items affected the results, we re-estimated the structural equation models using maximum likelihood with missing values (MLMV). The structural equation model estimates are identical (results available on request). However, as the MLMV method does not allow for the computation of key model fit statistics, we report the results based on a smaller sample after listwise deletion of missing values.

Using the continuous 24-point GEM scale as the dependent variable (instead of its four latent domains) produced qualitatively similar results, although the coefficients tended to be less precisely estimated. Table A2 in the Additional file 1 shows estimates from three regression models that differ in their assumptions regarding the nature of village-level heterogeneity, but all coefficients were of the same direction and comparable size.

## Discussion

This study analyses correlates of gender equitable attitudes among rural Tanzanian adolescents and reveals important dynamics around gender socialisation in four domains: domestic responsibilities, reproductive health, sexual relationships and violence.

Females were more likely to express inequitable attitudes across all but one of the 12 items of the GEM scale for which there was a statistically significant gender difference, including 4 out of 5 items in the ‘domestic chores’ domain. A gendered perspective of socialising female adolescents with more domestic chores may translate into female adolescents being less equipped to take care of themselves financially in adulthood. This has health-related implications, as a combination of financial dependency on men and gender inequitable attitudes is associated with females’ increased experience of violence and HIV risk behaviours such as transactional sex [2, 14–16].

Most of the adolescent girls in our sample were in school, but there are expectations that they engage in significant domestic chores [17], and this may mean that they have less time to study and their timely progression may be interrupted, limiting their educational attainment. The education curriculum in Tanzania requires students to pass national exams at different levels from primary, secondary to high school, and a failure at any of these levels means an end to schooling [18]. Thus, domestic chores may affect not only attendance, but time availability to prepare for these critical exams, impeding continuation of schooling. Previous research from sub-Saharan African countries showed that women with more schooling tended to delay sexual debut, with implications for related risks such as adolescent pregnancy [19]. Meanwhile, if boys are socialised to perform fewer household chores, not only do they have more time to study and possibilities to pursue higher levels of education, but they also have more choice to decide what they wish to do with their lives. Since boys are more likely to engage in economic activities in this setting [20], they are also more likely to transition into financially independent adults. Socialization into more equitable gender roles during adolescence is important for minimising cultural practices and beliefs that privilege boys and subordinate girls [21].

Our findings related to attitudes toward gender imbalances in household tasks is supported by a recent study implemented nationally in Tanzania using a version of the GEM scale [6]. Nevertheless, differences in age structures of the samples make it challenging to directly compare our findings with those in the national study, as the latter comprises only one-fifth of its sample with a similar age range (15–19 years) as our sample (14–19 years).

Gender equitable attitudes may change with age, education and experiences (including sexual debut). In fact, a previous study using the GEM scale in Uganda found that younger adolescents had more gender inequitable attitudes than older adolescents, suggesting that inexperience may lead younger individuals to ‘societal messages around relationships and gender at face value’ (page S20) [2]. This is in contrast to findings from our study, where we found younger adolescents to have more equitable attitudes in one of the districts, among two of the four domains examined (violence, and reproductive health). While our data do not allow us to understand what is driving the difference in age-related findings between the two contexts, it is possible that this difference is in part driven by differences in prevalence of sexual debut between the studies (19% in our study compared to 48% in the Ugandan study). Our findings do suggest that attitudes may evolve with personal experiences and transitions such as sexual debut, which correlate with increasing age. However, this effect was dependent on gender: sexual debut was associated with more gender equitable attitudes among females, but the reverse was true among males. Previous studies have shown that adolescent decision-making, including about sex, is heavily influenced by peer groups [22–24]. The influential power of the peer group could be harnessed in future interventions to tackle the issue of adolescent decision-making about sex and other important issues in more gender equitable ways.

Structural equation models provided stronger and more nuanced results than linear regressions with a 24-item GEM scale, allowing us to isolate the effects of each predictor on four latent domains. For example, in Mbeya, younger respondents expressed more equitable attitudes in the violence and reproductive health domains, with no significant age differences in the other two domains. Older respondents in this study are more likely to have sexually debuted or be in a relationship, and being in a cohabiting intimate relationship increases the risk of intimate partner violence as shown in another recent study from the Mbeya region [25]. Having some secondary education was associated with more equitable attitudes in the reproductive health and sexual relationships domains only (in Iringa). Schooling can influence gender attitudes both directly and indirectly. For example, gender socialization happens through curriculum, authority figures in schools influence adolescents’ perceptions of gendered roles, schools provide a context whereby adolescents form social networks in which peers can influence attitudes, and the act of going to school can also challenge traditional gender norms [1]. The household’s engagement in farm work was associated with adolescents’ attitudes regarding gender roles in the daily chores domain, and this may reflect daily reinforcement of gender-segregated tasks related to farming and household tasks.

### Limitations

Although this study is based on a large sample, it is drawn from rural, poor adolescents in four districts, limiting the generalizability of the results to all adolescents in Tanzania. Of our targeted sample, 1141 were not interviewed (31.7% of all eligible adolescents) and reasons included adolescents being unavailable for interview (some were temporarily away at school or visiting relatives). This may have influenced the representativeness of the study sample to the target population. In addition, responses on gender attitudes may suffer from social desirability bias. Finally, given the cross-sectional nature of the data, we cannot conclude causality between the relationships examined.

## Conclusion

The socio-ecological framework developed by John et al. proved useful in guiding the analysis of attitudes towards gender norms among rural adolescents in Tanzania [1]. Although the groups were relatively homogenous and socio-demographic characteristics explained only some of the differences in results, the framework allowed us to test determinants of gender attitudes in a holistic and organized way. Some of the determinants of the inequitable gender attitudes espoused by adolescents in the four districts could be explicitly addressed in the design of interventions. For example, a norm changing and life skills curriculum could discuss a healthy and gender-equitable approach to sex and intimate partnerships and promote benefits of education for girls. Given that girls had more inequitable gender attitudes than boys, a special emphasis on highlighting the individual rights of women, including to girls, should be considered. Further, given the fact that norms are passed on between generations, future interventions should consider engaging parents of adolescents.

## Supplementary information

**Additional file 1.**

## Data Availability

Data analysed for this study are not publicly available but may become available, subject to government approval, after completion of the impact evaluation.

## Abbreviations

CAPI: Computer-Assisted Personal Interview
CD: Coefficient of Determination
COSTECH: Commission for Science and Technology
cRCT: Cluster randomized control trial
GEM: Gender Equitable Men
MLMV: Maximum likelihood with missing values
OLS: Ordinary Least Squares
PSSN: Productive Social Safety Net
SRMR: Standardized root mean squared residual
TASAF: Tanzania Social Action Fund
