# Supplementary material for: Examining determinants of gender attitudes: evidence among Tanzanian adolescents
Source: BMC Womens Health. 2020 Sep 10;20:195. doi: 10.1186/s12905-020-01057-8 (PMC7488302; doi:10.1186/s12905-020-01057-8)
Supplement: Supplementary file 1 — Additional file 1. [file 12905_2020_1057_MOESM1_ESM.docx]

**Online Appendix**

**Appendix 1. Study Design and Sampling Information**

The impact evaluation (from which baseline survey data were used for the current study) uses a cluster randomized control trial (cRCT) design to examine impacts of an adolescent focused ‘Cash Plus’ intervention layered on top of Tanzania’s flagship social protection program, the Productive Social Safety Net (PSSN), implemented by the Tanzania Social Action Fund (TASAF). Ujana Salama complements the PSSN with a package of adolescent-focused interventions to strengthen productive, human and health capital to promote sustainable and healthy livelihoods that increase resilience, well-being and empowerment (6).

In this cRCT design, 130 clusters (communities) from two pilot administration areas of TASAF (in the Iringa and Mbeya regions of Tanzania) were randomized into two control and treatment arms, and randomization was stratified by district and village size (large v. small villages). During a public randomization event with district-level government officials, village names in each district were written on a piece of paper and placed into one of two hats (large villages v. small villages). District leaders then chose names from the hat and read them aloud, while the study coordinator wrote the village names down in order until all villages had been withdrawn. The top (first) half of the list was assigned heads and the second half tails. District officials completed a coin toss for each list (small and large villages) to assign villages to treatment.

All adolescents living in TASAF households in communities sampled within the eligibility age range were targeted for interviews, and sample size was determined based on power calculations of key outcomes from the study’s theory of change. Randomization took place in July 2017, after implementation of the baseline surveys (April – June 2017). The on-going evaluation is a multi-year, longitudinal, mixed-method study comprised of baseline (2017), wave 2 (2018), wave 3 (2019), and wave 4 (2020) surveys.

**Appendix Tables**

Table A1 Confirmatory factor analysis by district (four domains of the GEM scale)

|  |  | Mufindi (N=1,119) | Rungwe (N=761) |
| --- | --- | --- | --- |
| Violence | There are times a woman deserves to be beaten. | 0.40 | 0.48 |
|  | A woman should tolerate violence in order to keep her family together. | 0.43 | 0.51 |
|  | If someone insults a man he should defend his reputation with force if he has to. | 0.53 | 0.53 |
|  | It is okay for a man to hit his wife if she will not have sex with him. | 0.58 | 0.49 |
|  | A man using violence against his wife is a private matter that should not be discussed outside the couple. | 0.57 | 0.58 |
|  | It is alright for a man to beat his wife if she is unfaithful. | 0.55 | 0.53 |
| Reproductive health | It is a woman's responsibility to avoid getting pregnant. | 0.49 | 0.32 |
|  | A man should be angered/shocked if his wife asks him to use a condom. | 0.61 | 0.44 |
|  | Women who carry condoms on them are easy. | 0.48 | 0.33 |
|  | Only when a woman has a child is she a real woman. | 0.52 | 0.55 |
|  | A real man produces a male child. | 0.55 | 0.47 |
| Sexual relationships | It disgusts me when I see a man acting like a woman. | 0.25 | 0.27 |
|  | A woman should not initiate sex. | 0.51 | 0.51 |
|  | You do not talk about sex, you just do it. | 0.50 | 0.48 |
|  | A woman who has sex before she marries does not deserve respect. | 0.44 | 0.38 |
|  | Men need sex more than women do. | 0.62 | 0.48 |
|  | Men are always ready to have sex. | 0.55 | 0.49 |
|  | A man needs other women, even if things with his wife are fine. | 0.57 | 0.49 |
|  | It is the man who decides how he wants to have sex. | 0.57 | 0.54 |
| Domestic chores | Giving the kids a bath and feeding the kids are the mother's responsibility. | 0.62 | 0.60 |
|  | A woman's most important role is to take care of her home and cook for her family. | 0.60 | 0.61 |
|  | A man should have the final word on decisions in his home. | 0.64 | 0.67 |
|  | The husband should decide what major household items to buy. | 0.61 | 0.54 |
|  | A woman should obey her husband in all things. | 0.62 | 0.42 |
| Error covariances | Violence & Reproductive health | 0.817 | 0.672 |
|  | Violence & Sexual relationships | 0.736 | 0.557 |
|  | Violence & Domestic chores | 0.451 | 0.361 |
|  | Reproductive health & Sexual relationships | 0.892 | 0.779 |
|  | Reproductive health & Domestic chores | 0.707 | 0.613 |
|  | Sexual Relationships & Domestic chores | 0.703 | 0.573 |
|  | Standardized Root Mean Squared Residual (SRMR) | 0.048 | 0.045 |
|  | Coefficient of Determination (CD) | 0.976 | 0.975 |

All coefficients and error covariances are statistically significant at p<0.001. Variances are omitted. Standard errors adjusted for clustering by village.

Table A2 Predictors of equitable gender norms (GEM scale)

|  | Clustered standard errors (1) | | Village fixed effects (2) | | Village random effects (3) | |
| --- | --- | --- | --- | --- | --- | --- |
|  | Mufindi | Rungwe | Mufindi | Rungwe | Mufindi | Rungwe |
| Female | -0.99 | -1.86** | -0.74 | -1.75*** | -0.81* | -1.79*** |
| Age 14-15 | -0.01 | 0.76 | -0.13 | 0.90* | -0.15 | 0.84 |
| Some secondary education | 0.41 | 2.06*** | 0.67 | 1.72*** | 0.60 | 1.93*** |
| Ever had sex | 0.47 | -2.18** | 0.44 | -1.72* | 0.39 | -1.96** |
| Female * ever had sex | 0.73 | 1.88 | -0.05 | 1.57 | 0.19 | 1.78 |
| Farm work | 0.01 | -0.63 | 0.34 | -0.16 | 0.23 | -0.46 |
| Livestock herding | 0.14 | 0.47 | -0.44 | -0.03 | -0.30 | 0.31 |
| Household business | 1.05 | 1.48 | 0.39 | 1.24 | 0.62 | 1.38 |
| Distance to nearest daily market | -0.02 | -0.00 | -0.53** | -0.64* | -0.02 | 0.00 |
| Wife cannot inherit husband's land | 0.69 | 0.37 | 38.84** | 4.77* | 0.81 | 0.19 |
| Constant | 13.49*** | 11.98*** | 17.43*** | 20.96*** | 13.57*** | 11.82*** |
|  |  |  |  |  |  |  |
| N (individuals) | 1,030 | 668 | 1,030 | 668 | 1,030 | 668 |
| N (villages) | 66 | 51 | 66 | 51 | 66 | 51 |
| Intra-class correlation |  |  |  |  | 0.18 | 0.04 |
| R-squared | 0.02 | 0.08 | 0.23 | 0.18 |  |  |

*** p<0.001, ** p<0.01, * p<0.05
